# A bio-sustainable approach for reducing *Eucalyptus* tree-caused agricultural ecosystem hazards employing *Trichoderma* bio-sustained spores and mycorrhizal networks

**DOI:** 10.3389/fmicb.2022.1071392

**Published:** 2023-01-16

**Authors:** Md. Golam Kabir, Yonglong Wang, Md. Abuhena, Md. Faisal Azim, Jubair Al-Rashid, Noorain Munim Rasul, Dipa Mandal, Pulak Maitra

**Affiliations:** ^1^Department of Research and Development, Apex Biofertilizers and Biopesticides Limited, Gaibandha, Bangladesh; ^2^Institute of Soil Science, Chinese Academy of Sciences, Nanjing, China; ^3^International College, University of Chinese Academy of Sciences, Beijing, China; ^4^Faculty of Biological Science and Technology, Baotou Teacher's College, Baotou, Inner Mongolia, China; ^5^Apex Biotechnology Laboratory, Apex Holdings Ltd., Gazipur, Bangladesh; ^6^Tianjin Institute of Industrial Biotechnology, Chinese Academy of Sciences, Tianjin, China; ^7^Institute of Microbiology, University of Chinese Academy of Sciences, Beijing, China; ^8^Institute of Dendrology, Polish Academy of Sciences, Kórnik, Poland

**Keywords:** *Trichoderma* spp., *Serendipita indica*, *Eucalyptus*, rice, ecosystem hazards

## Abstract

The presence of the exotic *Eucalyptus* tree in crop-growing soil and the accumulation of its undecomposed leaves is a significant ecological hazard. The waxy coating on the leaves and the phenolic compounds takes a long time to break down under normal conditions. It is necessary to explore various fungi that can degrade these leaves for an eco-friendly solution to this problem. In this study, spores of nine native *Trichoderma* strains were produced on wheat agar using a lactic acid-induced sporulation strategy (LAISS). *Trichoderma* biosustained spores and *Serendipita indica* (SI) spores were applied to a rice field with accumulated *Eucalyptus* leaves under continuous ponding (CP) and alternate flooding and wetting conditions (AFW). Among the strains, TI04 (*Trichoderma viride*) and TI15 (*Trichoderma citrinoviride*) showed faster (5 days) and massive sporulation (1.06–1.38 × 10^11^ CFU/g) in LAISS. *In vitro*, TI04 and TI15 biosustained on *Eucalyptus* leaves and improved rice seedling growth and SI infection under greenhouse conditions. In the rice-field experiment, *Trichoderma*-treatment had a threefold yield (percentage) increase from control, with TI04 (CP) increasing the yield by 30.79, TI04 (AFW) by 29.45, TI15 (CP) by 32.72, and TI15 (AFW) rising by 31.91. Remarkably, unfilled grain yield significantly decreased in all *the Trichoderma* treatments. Under AFW conditions, TI04 and TI15 showed a higher pH increase. Furthermore, TI04 and TI15 under AFW had higher water productivity (t ha^−1^ cm^−1^) of 0.0763 and 0.0791, respectively, and the highest rates (percentage) of SI colonization of 86.36 and 83.16, respectively. According to the findings, LAISS-produced *Trichoderma* spores can be applied to break down persistent wastes and restore agricultural ecosystems through increased mycorrhizae networking.

## Introduction

Recent climate changes have increased the severity of environmental biotic and abiotic stresses and had widespread effects on global agricultural production. These stresses can be perceived as a worldwide challenge and are the main predicaments that hinder crop yield, value, total food security, and natural ecosystems. Field crop production confronts biotic and abiotic risks, such as droughts, floods, storms, and soil toxicity. Soil is the foundation of agriculture. All harvests for human nutrition and animal fodder rely on healthy soil. The effects of climate change have deteriorated the quality of this vital natural reserve. In addition to the vast amounts of anthropogenic garbage, the alien plant *Eucalyptus* and its accumulated leaves degrade soil quality. *The Eucalyptus* genus, with more than 800 species, is the most significant in the botanical family Myrtaceae. It is an Australian native and has an extensive global distribution (Hassine et al., 2013). It was brought to the region that is now Bangladesh in the 1930s. Going back in time, the tea estates in eastern Bangladesh carelessly introduced this tree as an ornamental species in the 1930s (Davidson and Das, 1985). When the Ministry of Environment and Forest department (MEF) discovered the negative impacts of *Eucalyptus* in 1995, they removed it from their tree planting program and made the public aware of those problems (Babu, 2018). They are aggressive species in Bangladesh because they can cause groundwater levels to drop and provide little benefit to native wildlife that prefers other tree species.

One of Eucalyptus’ main drawbacks is an extremely high rate of transpiration that causes detrimental effects on the environment. Compared to other trees in the country, *Eucalyptus* contributes much more to draught through transpiration 18–20 times higher. Groundwater levels may drop by 20% if *Eucalyptus*es are permitted to grow around a freshwater reserve for around 10 years, while other trees show growth at a rate of 10%. The *Eucalyptus* tree is more valuable economically, but it is also more invasive than other trees because its characteristics make it more attractive for planting (Babu, 2018). Unfortunately, because nurseries throughout the nation were producing and selling large numbers of these saplings, it was already too late to stop the out-of-control practice of *Eucalyptus* plantation. The environment is in grave danger as *Eucalyptus* plantations are currently so pervasive that they can be found in large numbers on almost every yard, office building grounds, by the side of the road, and along railroad tracks, especially in the northern parts of Bangladesh (Bogura, Gaibandha, Rangpur, Dinajpur, Panchagarh, Nilphamari, Thakurgaon, and Kurigram).

Environmental groups are concerned about the negative effects of *Eucalyptus* trees on the environment. These difficulties include high transpiration rates, declining soil fertility, incompatibility with the preservation of biodiversity, and allopathic impacts of *Eucalyptus* spp. that impede undergrowth regeneration (Aweto and Moleele, 2005; Cao et al., 2010; Cortez et al., 2014). Lane et al. (2004) in China discovered the development of *Eucalyptus* plantations lowers water tables and decreases the amount of water available for irrigation on lands previously used for crops or occupied by native trees and grasses due to the plant’s deep and extensive root system and soil hydrophobicity (Lane et al., 2004). Even the tree’s leaves are not amenable to easy decomposition and cause soil damage. *Eucalyptu*s planted as a crop with short rotation for high biomass production depletes soil nutrients rapidly. A biocide oil is present in *Eucalyptus* leaves. It prevents the majority of soil bacteria from surviving and decomposing the leaves. However, although it takes a little longer than with leaves of other types of trees, they do degrade. They require a little more time to dry out than other leaves because of the oil content. The availability of nutrients to the plants also depends on the degree of breakdown. There is a need to speed up the decomposition that is incorporated into the field due to the harmful environmental effects of undecomposed leaves and the consequences of crop production loss. The high water use of *Eucalyptus* trees also contributes to desertification in Bangladesh’s northern region, and ground water level drops harm natural agriculture ecosystems.

Microbial inoculants may assist in reducing such hazards by hastening the leaf breakdown process and relieving the plants from water-stressed conditions. *Serendipita indica* is a phyto-promotional, mutualistic root endosymbiont that can be grown axenically and imitates the capabilities of typical arbuscular mycorrhizal (AM) fungus (Narayan et al., 2021). This fungus has the ability to colonize the roots of a variety of higher plants and offers them numerous benefits (Unnikumar et al., 2013; Verma et al., 2021; Abuhena et al., 2022a). Additionally, *Serendipita indica* root endophyte increases plants’ tolerance and resistance to drought (Jogawat et al., 2016; Prasad et al., 2019). It effectively attaches to the root system and develops a vast root network of hyphal filaments that serve as extensions, outwardly extending the rhizosphere. It boosts a plant’s ability to absorb water, macronutrients, and micronutrients, and tolerate drought (Narayan et al., 2017).

Soil microorganisms play a part in the decomposition process since they are the ones that transform agricultural residues into soil organic matter. According to the study, the decomposition of rice straw is a multi-step, complex process that involves a variety of microbial species. The fungus *Trichoderma* is one of the most prevalent microorganisms that degrade rice straw. *Trichoderma* sp. is classified as a decomposer fungus due to its saprophyte nature, and as such, its function is to provide free organic materials, particularly during the supply process of compost. Prior studies have suggested that *Trichoderma* spp. may speed up the breakdown of leaves (Organo et al., 2022). According to reports, *Trichoderma* spp. may quickly break down household and agricultural waste without producing unpleasant odors. With the aid of a cellulase enzyme, *Trichoderma* spp. can break down leaves that include components such as cellulose (Organo et al., 2022). *Trichoderma* spp. can produce enzymes like cellulase, cellobiose (β-Glucosidase), and chitinase. As the cellulase enzyme is a multi-enzyme made up of cellobiohydrolase, endoglucanase, and β-glucosidase, it is an enzyme that takes part in the breakdown of organic matter. *Trichoderma* can enhance crop output, resistance to abiotic stresses, uptake and use of nutrients, and root growth and development (Sood et al., 2020). Throughout the growth phase, it can cause systemic resistance in plants (Nawrocka and Małolepsza, 2013). *Trichoderma* isolates infiltrate the rhizosphere of plants and increase the variety of the soil microbiome (Saravanakumar et al., 2017). The lack of highly efficient spores for these organisms restricts novel applications, besides the potential for *Trichoderma* strains in boosting plant health and decomposition. *Trichoderma* is most frequently grown on conventional solid media. Solid-state fermentation (SSF) produces more resilient and active spores with a longer lifespan, and higher resistance (Naeimi et al., 2020). However, spore concentration and stability are typically poor, and contaminations occur frequently during liquid fermentation operations. Semi-solid fermentation can generate healthy spores. As a result, novel techniques utilizing various alternative cultivation conditions and nutrients can produce bio-sustaining spores of *Trichoderma*.

Therefore, a combined strategy was adopted here by applying *Trichoderma* fungi to accelerate the decomposition of the *Eucalyptus* leaves in the low pH soil of a rice field. In particular, we produced stress-tolerated *Trichoderma* spores using lactic acid mixed wheat agar to induce the spores’ biosustaining potential. Furthermore, the improvement in the rice field ecosystem was fostered by mycorrhizae networking, minimizing the water scarcity hazard caused by *Eucalyptus* trees.

## Materials and methods

### Fungal and plant materials

Apex Biotechnology Laboratory provided the endophytic *Serendipita indica* (ATCC^®^ 204458TM) culture, which was obtained from ATCC, University Boulevard Manassas, United States. The rice cultivar (BRRI Dhan29) utilized in this study was supplied by the Bangladesh Rice Research Institute (BRRI).

### SI inoculum preparation

To prepare the inoculum, SI was first grown on M-media (Maitra et al., 2021) in a petri dish, and then it was transferred to the seed culture of Kaefer medium (Hill and Kafer, 2001) by cutting out 8 mm of agar discs. The seed culture was grown for 10 days at 30°C on a rotary shaker in a 500-mL flask with 100 mL of medium (Biobase, China).

### Isolation of *Trichoderma* spp.

For the isolation of *Trichoderma* spp., environmental samples were collected from different agro-ecological zones of Bangladesh (Supplementary Table 2). Upon collection, the samples were taken to the Apex biotechnology laboratory (ABL) in Gazipur, Bangladesh as soon as possible and securely maintained in a refrigerator at 4°C before being immediately analyzed. According to Abuhena et al. (2022b), a dilution plate approach was used to isolate *Trichoderma* spp.

### Molecular characterization of *Trichoderma* spp.

According to Abuhena et al. (2022b), the PrepMan^®^ Ultra Sample Preparation Reagent Kit (Applied Biosystems, United States) was used to isolate genomic DNA from a single colony of a Trichoderma isolate. PCR was performed in a MyGeneTM Series Peltier Thermal Cycler using genomic DNA isolated from a Trichoderma isolate and a positive control of Saccharomyces cerevisiae with standard primers provided in the Fast MicroSEQ^®^ D2 LSU rDNA Fungal PCR Kit (Applied Biosystems, United States). The MicroSeq^®^ D2 LSU rDNA Fungal Sequencing Kit’s Forward Primer Reaction Mix and purified PCR product were used in cycle sequencing (Applied Biosystems, United States). Using the NCBI BLAST system,^1^ DNA similarity was examined. To identify species, a criterion of 98% sequence similarity was applied. The isolated strains’ D2 LSU rDNA sequences were added to the NCBI GenBank database and given an accession number. The phylogenetic analyses were conducted in MEGA11.0 (Maitra et al., 2021).

### Antagonistic activity against phytopathogens

According to Abuhena et al. (2022b), *Trichoderma* isolates were subjected to *in vitro* tests to measure their potential to prevent the growth of phytopathogenic fungi. *Sclerotium delphinii* SCR5 (MK478832), *Fusarium equiseti* PTR3 (MK478826), *Curvularia spicifera* BLR4 (MK478825), and *Alternaria alternata* ALT10 (MK478825) were the four fungal phytopathogens (CCN: 110–113) provided by ABL (Apex Biotechnology Laboratory) and utilized in this investigation. Two 5 mm borer holes, spaced 6 cm apart and 10 mm from the edge of each sterile PDA plate, were drilled into each plate. Utilizing cultures of both *Trichoderma* and Phytopathogen isolates that were 5 days old, *in vitro* antagonistic assays were set up. Using sterile forceps, *Trichoderma* and plant pathogen culture discs (5 mm in size) were inserted into the holes of each dish from the opposite direction. Dishes with only the discs of the pathogens were used as controls. The growth inhibition of phytopathogens by *Trichoderma* was measured after 7 days of incubation at 28 ± 2°C using the following formula (Equation 1):


(1) I=(C−T)/C×100

Where I stands for the percentage of the phytopathogens’ growth inhibition, C for the radial growth of the phytopathogens (in cm) in the control plate, and T for the radial growth of the phytopathogens (in cm) in the test Petri plates.

### Quantitative estimation of indole acetic acid production, phosphate (P) solubilization and Chitinase and β-1, 3-Glucanase activity

IAA output was calculated according to Abuhena et al. (2022c). Three 5 mm plugs were inoculated in potato dextrose broth (PDB) supplemented with 0.2% filter-sterilized (Whatman) L-tryptophan solution from 5-day-old *Trichoderma* cultures and incubated at 28°C in a rotary shaker at 120 rpm for 7 days. The quantity of IAA in the culture was measured in micrograms per milliliter. On the other hand, quantitative estimation of phosphate solubilization was carried out using Erlenmeyer flasks (100 mL) containing 50 mL of medium inoculated in triplicate with three discs (5 mm in diameter) of actively growing cultures of *Trichoderma* isolate following Abuhena et al. (2022c). Phosphate in the culture supernatant was estimated and expressed as equivalent phosphate (μg/mL). Likewise, according to Abuhena et al. (2022c), the activities of Chitinase and β-1, 3-Glucanase were assessed using the modified *Trichoderma* liquid enzyme (TLE) medium.

### Media for *Trichoderma* cultivation and its spore production

Potato dextrose agar (PDA; HiMedia) and wheat agar (WA) media were utilized in this experiment. The PDA media contained potato infusion (200 g/L), dextrose (20 g/L) and agar (20 g/L), whereas the WA media contained wheat flour (20 g/L) and agar (22 g/L). The wheat was collected from the BARI (Bangladesh Agricultural Research Institute) regional center in Rajshahi, Bangladesh. The wheat was cleaned, dried, and stored in a dry place in the laboratory until utilization. Moreover, wheat grains were milled using an automatic grain flour milling machine (Kingrunda, model: 6FT-28), which yielded 0.08 mesh flour.

### Seed suspension preparation

To prepare a seed suspension, *Trichoderma* isolate was revived from a glycerol (20%) stock in a PDA plate. The retrieved culture was then sub-cultured twice. A single colony was seeded on PDA plates (11 cm in diameter) from the second subculture and incubated at 28 ± 2°C with pH 5.8 ± 0.2. After 5 days of incubation, spores were scraped from the Petri plate’s surface and placed in sterile DM (demineralized) water containing 0.9% NaCl, then shaken in the incubator (Biobase B110) for 2 h. After shaking, the spore suspension was filtered through cheesecloth to prevent mycelia from clogging the sprayer nozzle during inoculation. A seed suspension was prepared with a concentration of 1 ± 0.3 × 10^5^ CFU/mL (Abuhena et al., 2022c).

### *Trichoderma* spores production using pH-dependent nutrient deprivation approach

The manufacture of seed cultures was accomplished 5 days ahead of the inoculation of the production media. In the pH-dependent nutrient deprivation experiment, PDA media was used as a control and WA as a production medium for the manufacturing of spores of nine *Trichoderma* isolates. Agar at a concentration of 22 g/L was added to the media to harden it and allow just the spores to be harvested by scrapping without taking any media residue, and lactic acid was added at a concentration of 1 (mL/L) to induce sporulation and suppress contamination. The WA medium was first properly mixed at 60°C to avoid coagulation prior to sterilization. After sterilizing, the medium was thoroughly mixed again just before pouring. The media (3 L) was then poured into the sanitized stainless-steel tray. After 1 h of medium pouring, the media was inoculated with a 2% seed suspension using a hand sprayer. The inoculated media was incubated to produce a substantial, deep-green spore mat in the dark. The spores were scraped from the media surface without getting any media residue after 5–7 days of inoculation.

### Biosustaining potential of *Trichoderma* spores on *Eucalyptus* leaves *in vitro*

Following harvesting, the spores of different *Trichoderma* isolates produced on WA media were evaluated for their biosustainability and growth on *Eucalyptus* leaves. After washing with sterile distilled water (1–2 times), the *Eucalyptus* leaves were surface sterilized by dipping in 3% sodium hypochlorite (5 min), followed by dipping in 95% ethanol (20 s; from dipping to washing with distilled water), and finally washing 5 times with sterile distilled water. There was 5 g of dried leaf in each plate. *Trichoderma* spore suspension (1 mL) diluted with 19 mL of water was mixed with leaves as inoculation. There were 10 treatments: T1-T9 for *Trichoderma* spores treated leaves (for nine isolates) and T10 for non-treated leaves (control). For each treatment, five replications were used in this experiment. All treatments were incubated at 30 ± 2°C and 75 ± 2% relative humidity in an incubator (BIOBASE: BJPXA450). After 15 days, the growth of each isolate was observed and data was recorded.

### Rice seedling growth assay

In a seedling growth assay, rice (*Oryza sativa*) was used to demonstrate the growth-promoting potential of biosustained *Trichoderma* strains in greenhouse conditions (Abuhena et al., 2022b; Xue et al., 2021). After washing with autoclaved water (1–2 times), the seeds were disinfected by dipping in 75% ethanol (1 min), followed by dipping in 0.5% sodium hypochlorite (5 min), and finally washing 5 times with autoclaved water. After 1 h of autoclaving at 121°C and 15 pressure, the soil was collected (Abuhena et al., 2022d). Soil was mixed with 0.5 g of degraded leaf-mixed *Trichoderma* spore and kept overnight. There was 250 g of dried soil in each plastic cup. In each cup, three seeds were sown after 48 h of soaking with *Serendipita indica* (SI) inoculum. There were 11 treatments: T1–T9 for *Trichoderma* spores and SI application (for nine isolates) and T10 for non-treated seedlings (control). T11 (sole SI) was also used as control for checking SI infection rate in rice root. For each treatment, five replications were used in this experiment. For up to 30 days, all plants were grown in a greenhouse at 28 ± 2°C and 75 ± 5% relative humidity. Additional plants were discarded from the cups at 5 DAG (Days after germination) while keeping one plant in each cup. After 30 DAG, seedlings were harvested from each treatment for data collection.

### Application of biosustained spore in rice fields with accumulated *Eucalyptus* leaves

A field experiment was conducted in the research field of ABBL (Apex Bio-fertilizer & Bio-pesticides Ltd., Gobindaganj, Gaibandha, Bangladesh: 25° 148′N, 89°89′) to determine the impact of TI04 and TI15 spores produced by LAISS and formulated with 2 percent falvic acid on the yield and yield-related parameters of rice (BRRI Dhan29) through the decomposition of *Eucalyptus* leaves and increasing soil quality. A Randomized Complete Block Design (RCBD) with five replications was used to set up the experiment (Supplementary Figure 4). The following treatments were used: T1–TI04 (CP: continuous ponding), T2–TI04 (AFW: alternate flooding and wetting at 1 day after disappearance of 4 cm), T3–TI15 (CP), T4–TI15 (AFW), T5-control (CP), and T6-control (AFW). The formulated *Trichoderma* spore was applied with a compost (dose: 4 kg/ha) applied in the soil before planting and SI as seedling dipping (dose: 5 mL/L). The fertilizer dosage was applied in accordance with SRDI (Soil Resource Development Institute, Bangladesh). Rice seedlings were planted in mid-February. The line-to-line distance in each plot was 20 cm, and the plant-to-plant distance was 15 cm. Irrigation was given to provide 4 cm of surface water after the plants were established (around 3 weeks after transplantation), and then irrigation was provided according to treatments T1, T2, T3, T4, T5, and T6. When necessary, the specific plot was wet. The relevant data was collected both during and following the experiment. The amount of irrigated water used was tracked from 15 days after transplanting (DAT) to 15 days before harvest. By dividing the overall yield by the total amount of water needed throughout the full crop growth period, the water usage efficiency (WUE) of rice was determined (Hasan et al., 2019):

Water use efficiency = Y/WR (t ha^−1^ cm^−1^)
Where, Y = grain yield (t ha^−1^), WR = total amount of water used (cm)
Analytical technique

The spore density was determined using the spread plate technique. Dilution factors from 10^−1^ to 10^−1^ were used to dilute cell suspensions. CFU were counted after 48–72 h of incubation at 28 ± 2°C on PDA plates.

### Assessment of *Serendipita indica* colonization

The root sample was taken and rinsed with demineralized water after 65 days of rice seedling planting. The root segments (1 cm long) were softened in 10% potassium hydroxide at 90°C for 15 min to facilitate de-colorization. To determine the fraction of root length colonized by *Serendipita indica*, the sample was neutralized for 5 min at room temperature with a 2% HCl solution and stained for 2 min at 90°C with a 2% trypan blue-lactoglycerol solution dye. The root colonization percentage was calculated using the magnified intersection method (Maitra et al., 2021).

### Statistical analysis

XLSTAT (2021) was utilized to carry out the PCA (XLSTAT, 2021). Using R program (R Development Core Team, 2021), One-way ANOVA was conducted to test the influence of *Trichoderma* spores with SI on grain yield and water productivity under different cultivation conditions. A pairwise comparison by *t*-test was conducted to test the effect of *Trichoderma* spores on pH change before and after inoculation. Tukey’s honestly significant difference (HSD) test was used to examine the significant difference of different *Trichoderma* isolates on bioactivity, *Serendipita indica* colonization in rice seedling roots, and seedling growth parameters of rice if the data satisfied the normality of distribution and homogeneity of variance after log or square root transformation. For the data that did not satisfy the normality of distribution and homogeneity of variance after transformation, a nonparametric Conover’stest using the ‘*post hoc*.kruskal.conover.test’ function in the PMCMR package (R Development Core Team, 2021).

## Results

### Isolation and identification of *Trichoderma* spp.

**Figure 1:**
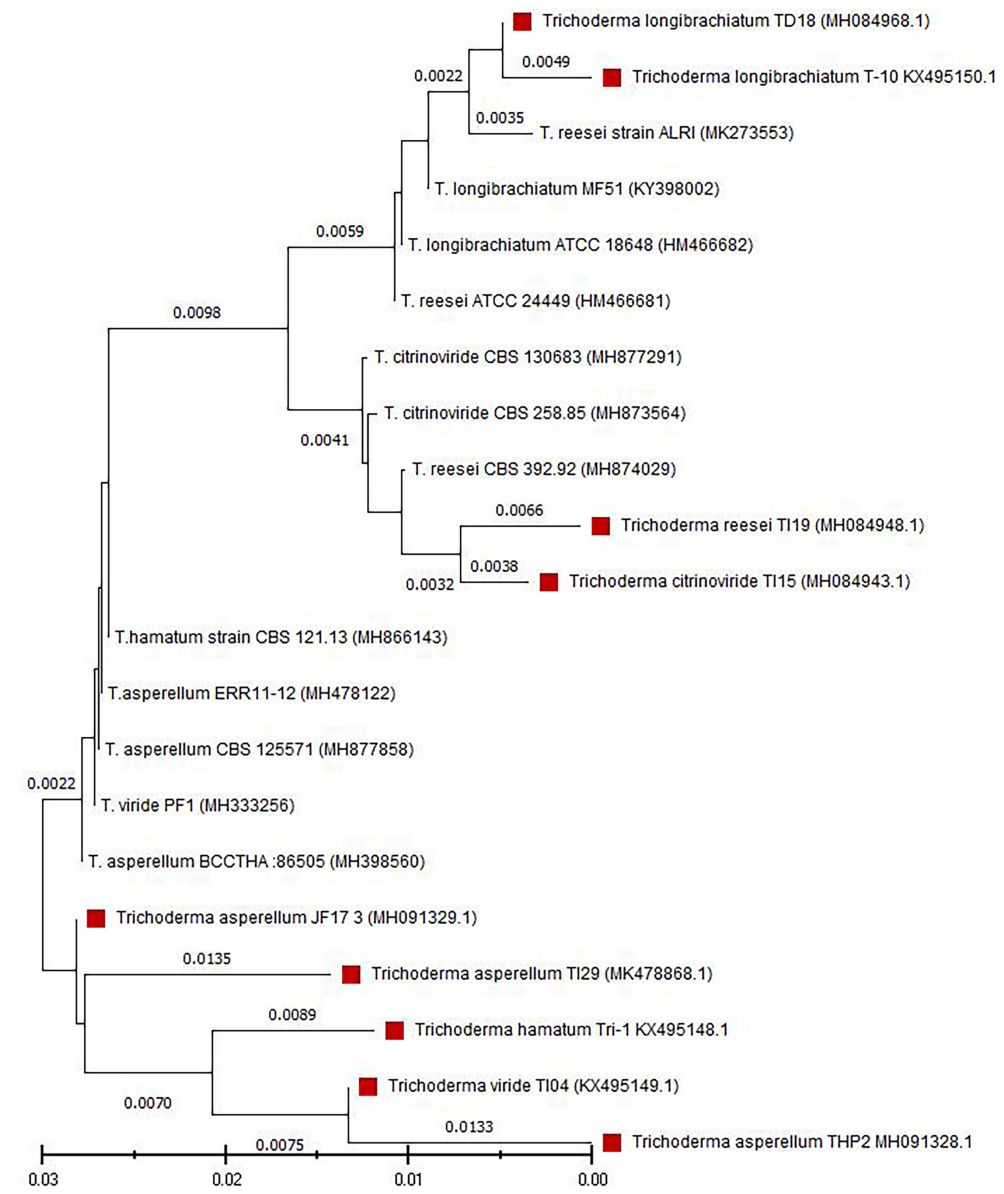
Phylogenetic analysis of nine *Trichoderma* isolates. Tree was constructed by the neighbor-joining method. The numbers given over branches indicate bootstrap coefficient.

Nine fungal isolates were isolated from a variety of samples collected from different agri-ecological zones of Bangladesh. These strains have deep green colonies with a smooth edge and watery white floccose mycelial growth (Supplementary Figure 1). Conidia were ellipsoidal and subglobose in shape and densely branched. Phialides were flask-shaped and diverged into 2–4 whorls (Supplementary Table 1). The strains were identified as six different *Trichoderma* species using D2 LSU rDNA sequences, including *Trichoderma viride* (TI04), *T. citrinoviride* (TI15), *T. reesei* (TI19), *T. hamatum* (Tri1), *T. longibrachiatum* (T10 and TD18), and *T. asperellum* (TI29, THP2, and JF17-3). Figure 1 depicts the outcomes of a phylogenetic analysis of nine *Trichoderma* isolates.

### Bioactivity of *Trichoderma* isolates

*Trichoderma* isolates revealed substantial differences in growth suppression against four different phytopathogens (Table 1). Isolate TI04 showed the highest inhibition effect on the growth of *Sclerotium delphinii* SCR5 (71.32%), *Fusarium equiseti* PTR3 (63.08%), *Curvularia spicifera* BLR4 (58.95%), and *Alternaria alternata* ALT10 (56.53%; Supplementary Figure 2). The isolate TI15 showed the second greatest inhibitory effect on phytopathogen growth. THP2, on the other hand, inhibited phytopathogens the least. The majority of the isolates demonstrated inhibition values ranging from 40% to 60% against phytopathogens. Chitinase and β-1,3 glucanase were produced by all of the *Trichoderma* isolates used in this study. All of the isolates secreted chitinase and β-1,3 glucanase at concentrations ranging from 0.39% to 3.08 units/mL and 0.33 to 1.95 units/mL, respectively, as shown in Table 1. The highest chitinase activities were found in TI04 (3.08 U/mL), TI15 (2.62 U/mL), T10 (2.49 U/mL), and Tri-1 (1.22 U/mL). The highest β-1, 3-glucanase activities were found in TI04 (1.95 U/mL), TI15 (1.63 U/mL), T10 (1.48 U/mL), and Tri-1 (1.07 U/mL). Chitinase (0.39 U/mL) and β −1,3-glucanase (0.33 U/mL) activities were lowest in THP2. On the other hand, the majority of the isolates exhibited moderate chitinase and β-1,3-glucanase activity.

**Table 1:** Bioactivity of *Trichoderma* isolates: antagonistic activity against phytopathogens, Chitinase and β-1,3-glucanase activity, phosphate solubilization and IAA production.

*Tricho-derma* isolates	Antagonistic activity against Phytopathogens				Chitinase (Units/mL)	β-1,3-Glucana se (Units/mL)	P concentration (μg/mL)	IAA concentration (μg/mL)
	SCR5	PTR3	BLR4	ALT10				
TI04	71.32 ± 3.64 a	63.08 ± 5.66 a	58.95 ± 4.45 a	56.53 ± 4.70 a	3.08 ± 0.13 a	1.95 ± 0.10 a	361.02 ± 20.41 a	38.03 ± 2.77 a
TI15	65.00 ± 7.44 a	59.27 ± 1.16 ab	59.00 ± 3.45 ab	52.43 ± 5.64 a	2.62 ± 0.08 b	1.63 ± 0.12 b	241.33 ± 18.37 b	16.07 ± 4.69 bc
TI19	48.19 ± 4.13 b	47.85 ± 5.35 bc	46.16 ± 3.43 ab	47.43 ± 4.76 a	0.77 ± 0.16 de	0.39 ± 0.05 e	115.38 ± 21.69 fg	2.63 ± 1.27 d
TI29	57.07 ± 5.42 a	45.96 ± 2.19 bc	48.50 ± 2.81 ab	44.53 ± 2.59 a	0.93 ± 0.07 cd	0.64 ± 0.04 d	162.93 ± 7.94 de	4.90 ± 2.70 d
T10	63.19 ± 3.63 a	51.45 ± 7.34 abc	54.84 ± 3.47 ab	48.99 ± 4.52 a	2.49 ± 0.08 b	1.48 ± 0.06 b	227.16 ± 18.21 bc	19.74 ± 5.20 b
Tri-1	63.15 ± 4.73 a	48.81 ± 4.25 bcd	45.56 ± 7.58 b	48.72 ± 3.08 a	1.22 ± 0.10 c	1.07 ± 0.07 c	185.69 ± 10.08 cd	10.76 ± 3.90 bcd
THP2	49.97 ± 6.05 b	44.63 ± 3.39 c	44.76 ± 8.07 b	47.53 ± 4.32 a	0.39 ± 0.06 f	0.33 ± 0.05 e	132.47 ± 7.95 efg	7.46 ± 5.42 cd
JF17-3	56.68 ± 6.76 a	47.23 ± 5.63 bc	48.08 ± 4.14 ab	45.87 ± 3.47 a	0.66 ± 0.07 def	0.68 ± 0.08 d	154.28 ± 6.56 def	4.31 ± 1.24 d
TD18	56.58 ± 11.3 a	48.10 ± 5.18 bc	48.62 ± 4.93 ab	46.05 ± 4.85 a	0.61 ± 0.08 ef	0.52 ± 0.09 de	134.81 ± 22.86 efg	2.92 ± 1.75 d

Data (means ± SE, *n* = 5) in the same column with different letters are significantly different at *p* < 0.05 by Tukey’s HSD test or Conover test.

Isolates TI04 (38.03 g/ml) and T10 (19.74 g/ml) produced the highest IAA, while isolates TI19, TD18, JF17-3, and TI29 produced the lowest (range 2.63–4.90 g/mL; Table 1). On the other hand, IAA levels in the isolates TI15, Tri-1, and THP2 were moderate, ranging from 7.46 to 16.07 (g/mL). In NBRIP broth, all of the cultures showed good mycelia development, with TCP disappearing at the same time. Phosphate concentrations in culture filtrates from *Trichoderma* spp. ranged from (μgmL^−1^) 115.38 to 361.02 (Table 1). In the culture filtrate, the isolate TI04 had the highest phosphate concentration (361.02 gml^−1^), followed by TI15 (241.33), and T10 (227.16). The phosphate content (μgmL^−1^) of culture filtrates from TI19 (115.38) and THP2 (132.47) was significantly reduced after 96 h. Moreover, in all of the cases, a steady fall (4.1–5.8) in pH levels was seen.

### *Trichoderma* spores production using pH-dependent nutrient deprivation approach

**Figure 2:**
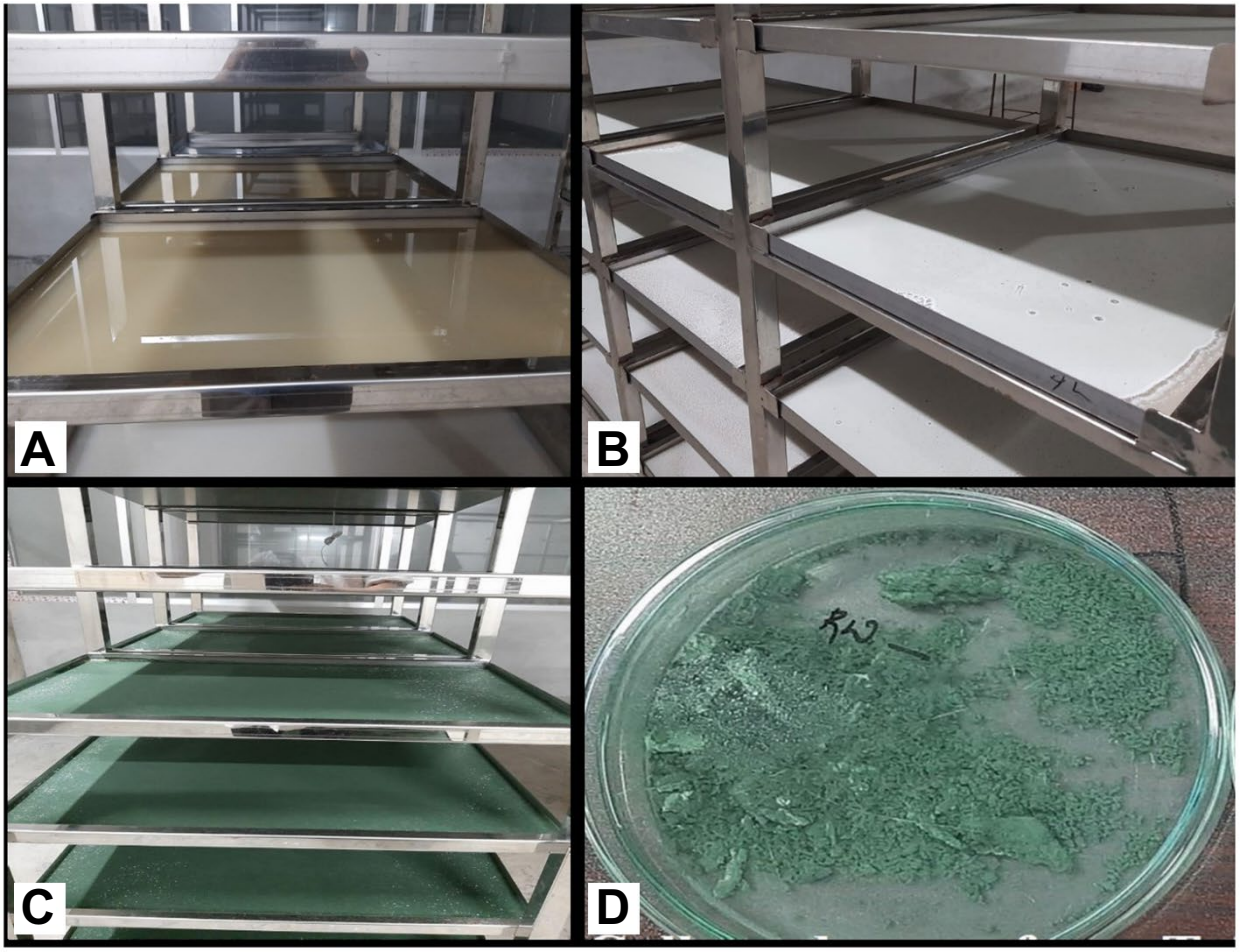
Sporulation of *Trichoderma* isolates on WA medium employing LAISS. **(A)** Media pouring, solidification, and spore suspension inoculation. **(B)** Visual growth of white mycelia **(C)** Spore growth appears as a green layer. **(D)** Collected spore.

In this approach, the spores germinated after spraying the spore suspension on the media (WA) surface, and the hypha that emerged from the spore lengthened at the terminal. More branches sprouted along the hypha. The branches continued to branch, forming mycelium and a porous three-dimensional network of hyphae. Initially, sparse mycelia were grown on the surface, then randomly penetrated the media because of its low water activity tolerance and ability to excrete bioactive compounds (Carlsen et al., 1996). During semi-solid fermentation, *Trichoderma* has a unique morphology that allows it to colonize and penetrate the semi-solid media surface in quest of nutrients. Mycelia continued to grow, and as a result, the media (WA) surface was completely covered by mycelia by day 2. On the third day, sporulation began at the surface, which quickly became dense after day 5 (Figure 2). Because the PDA medium (control) included the readily available nutrient dextrose, it had contaminant growth on the surface, whereas the WA medium had no contaminant growth. In PDA, all the isolates showed less spore yield of 1.72–2.03 g/L with a density of 5.24–7.86 × 10^9^ CFU/g. *Trichoderma* has the ability to produce a starch-degrading enzyme (α-amylase) that allows it to use starch (WA; Mohamed et al., 2011). *Trichoderma* can also obtain ATP through the metabolism of fiber material present in wheat flour. Some *Trichoderma* species produce incredibly effective siderophores that complexes iron and prevent the growth of other fungi (Benítez et al., 2004).

**Figure 3:**
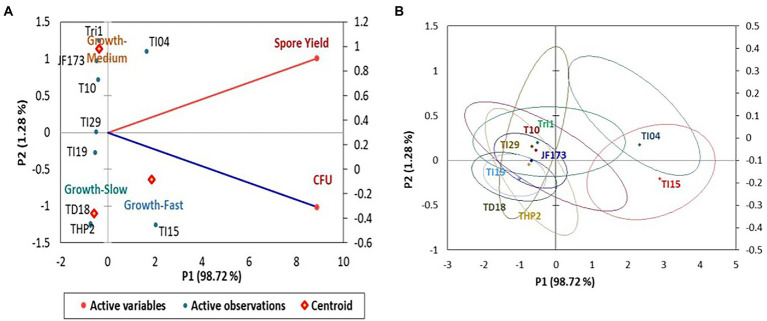
PCA plot of the growth pattern of nine *Trichoderma* isolates under LAISS. **(A)** A biplot of spore yield and density. **(B)** Bootstrap ellipses based on spore density and yield.

In the experiment, isolate TI15 had a significantly greater spore yield of 5.90 ± 0.02 g/L with a higher density of 1.38 × 10^11^ CFU/g, whereas TI04 had a second highest spore yield of 5.75 ± 0.14 g/L with a density of 1.06 × 10^11^ CFU/g. On the other hand, strain THP2 produced the lowest amounts of spores (2.86 ± 0.22 g/L) with a density of 3.26 × 10^9^ CFU/g. A biplot and bootstrap ellipses were constructed in PCA to depict the growth pattern of *Trichoderma* isolates (Figure 3). The first two principal component axes, PC1 and PC2, respectively, accounted for 98.72% and 1.28% of the overall variation in CFU and spore production by different *Trichoderma* isolates. The first and second components’ Eigen values and variability (%) were 1.974, 0.026 and 98.719, 1.281, respectively. All *Trichoderma* isolates were divided into three groups in four quadrants: “fast growers,” which required 5 days, “medium growers,” which required 6 days, and “slow growers,” which required 7 days. The first group contained the isolates from quadrant one and two (right upper and lower), which were *Trichoderma* isolates having a high spore-producing ability with high CFU content. It involved TI15 and TI04, which showed higher growth under LAISS. The second category was comprised of isolates from quadrant 3 (upper left), which contained Tri-1, T10, and JF173, the medium spore producers. The third group consisted of *Trichoderma* isolates with low spore generation and CFU, such as TI19, TI29, THP2, and TD18 (lower left).

### Biosustaining potential of *Trichoderma* spores on *Eucalyptus* leaves *in vitro*

**Figure 4:**
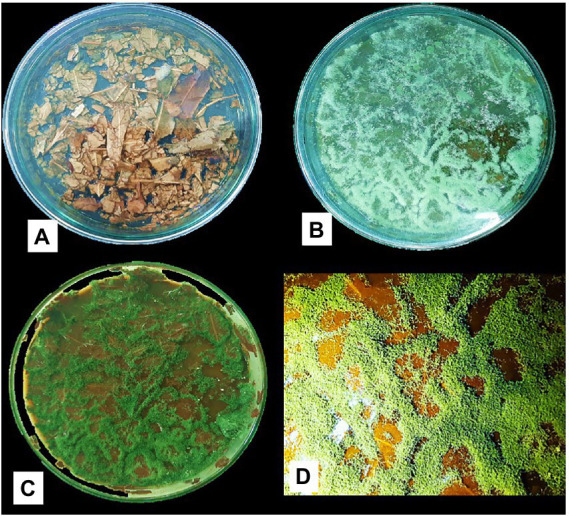
Growth of *Trichoderma* isolate (TI04) on *Eucalyptus* leaves **(A)** Before inoculation with spore suspension **(B)** Growth after 6 days of inoculation **(C)** Growth after 15 days of inoculation **(D)** Scattered spore mat on degraded *Eucalyptus* leaves.

**Figure 5:**
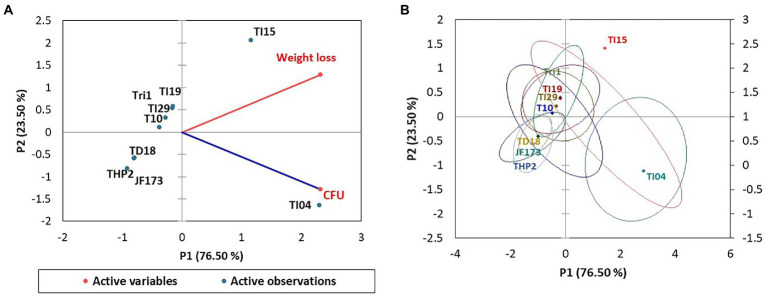
PCA plot of the growth pattern of nine *Trichoderma* isolates on *Eucalyptus* leaves. **(A)** A biplot of spore density and leaves weight loss. **(B)** Bootstrap ellipses-based spore density and leaves weight loss.

Figure 4 shows the *Trichoderma* growth for the 15 days of a laboratory experiment utilizing *Eucalyptus* leaves. In the first 6 days, white thread like mycelial growth were observed. After that, the sporulation of *Trichoderma* strains appeared. The overall growth of different *Trichoderma* strains was varied on *Eucalyptus* leaves. In the experiment, isolate TI04 had a significantly greater growth of 5.26 × 10^8^ (CFU/g of degraded leaf sample), whereas TI15 had the second highest growth of 7.05 × 10^7^ CFU/g (Figure 5). TI19, JF17-3, and TD18 produced the lowest number of spores (3.54 × 10^5^–4.55 × 10^5^ CFU/g; Figure 5). On the other hand, the CFU of the isolates THP2, Tri-1, T10, and TI29 were moderate, ranging from 1.46 × 10^6^ to 5.36 × 10^6^ (CFU/g). In the case of leaf degradation, isolates TI15 and TI04 showed maximum biodegradation with 31.33% and 26.8% weight loss, respectively, whereas isolates THP2 and JF173 showed the lowest biodegradation with 12% and 14.13% weight loss, respectively. In PCA, a biplot and bootstrap ellipses were used to depict the growth pattern of *Trichoderma* isolates on *Eucalyptus* leaves (Figure 5). PC1 and PC2, the first two principal component axes, attributed for 76.50 percent and 23.50 percent of the overall variation in CFU and weight loss by different *Trichoderma* isolates, respectively. Eigen values and variability (percentage) for the first and second components were 1.530, 0.470 and 76.498, 23.502, respectively.

### Rice seedling growth assay

**Figure 6:**
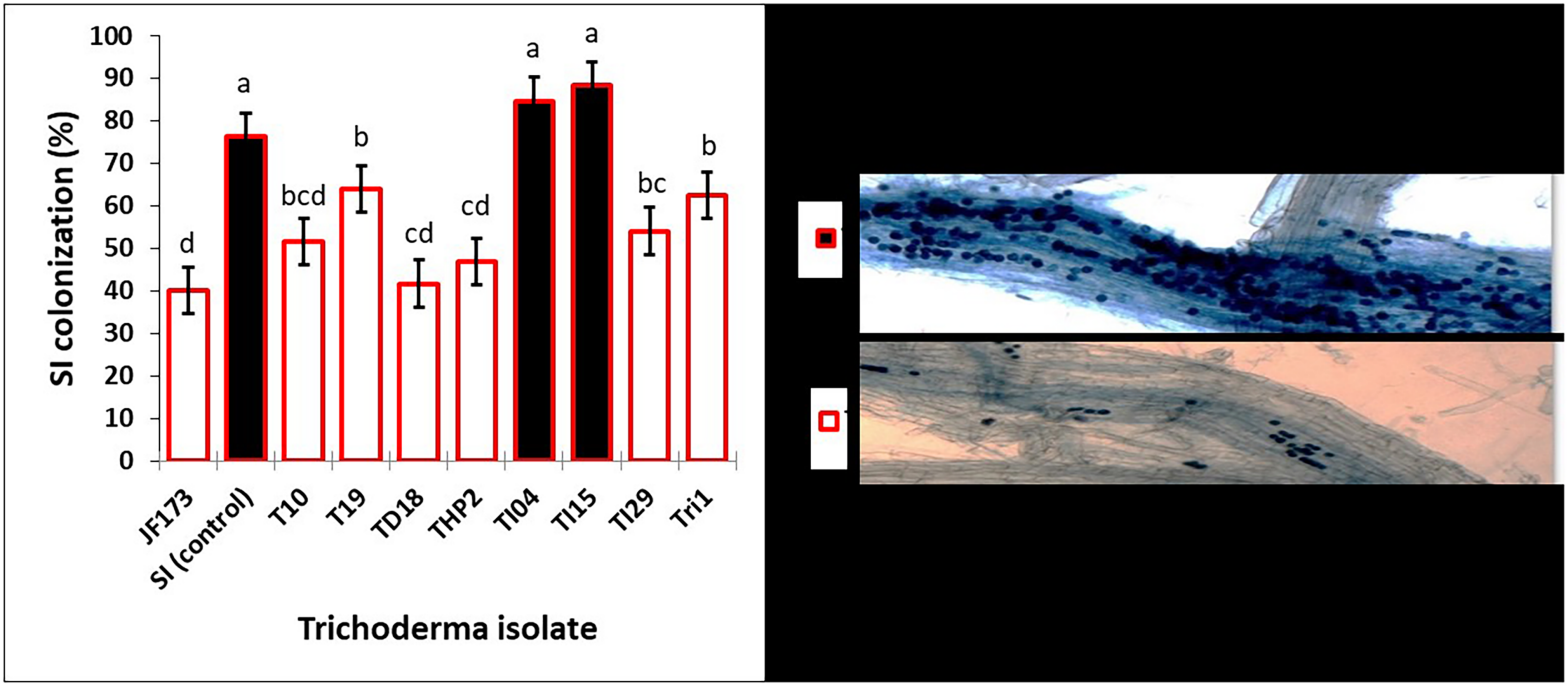
Effects of various degraded leaf-mixed *Trichoderma* spores on *Serendipita indica* colonization in rice seedling roots. Data are means ± SD (*n* = 5). Bars without shared letters indicate significant differences according to Tukey’s HSD test.

The seedling growth assay results in Table 2 show that degraded leaf-mixed *Trichoderma* spores improve shoot length (cm), root length (cm), and biomass of rice seedlings when compared to the control. Strains TI04 and TI15 outperformed the control in terms of shoot length (cm), root length (cm), and biomass. While strains TI29, T10, and JF17-3 significantly increased shoot length (cm) over controls, root length (cm) and biomass improvement were not significant. Since the leaves vary in available nutrients based on degradation percentage, the root association of SI mycorrhizae was shown to differ significantly between treatments. The rates (percentage) of SI colonization in seedlings treated with TI15 and TI04 were 88.42 ± 5.67, and 84.75 ± 9.30, respectively. These rates were significantly higher than those observed in seedlings treated with other isolates of Tri-1, T19, TI29, T10, THP2, TD18, and JF17-3 (ranges 40.13%–63.87%). In solely SI treated seedlings, the colonization rate was 78.25 ± 8.40%, which was slightly lower as compared to TI15 and TI04 but significantly higher than most of the isolate treated seedlings. This suggests that other *Trichoderma* isolates with less degraded leaves inhibit the infection of SI, whereas TI15 and TI04 with more degraded leaves have a synergistic effect on SI (Figure 6). However, no colonization was seen in the control seedlings.

**Table 2:** The influence of *Trichoderma* isolates with SI on seedling growth of rice under greenhouse conditions.

Isolates	Rice		
	Shoot length (cm)	Root length (cm)	Biomass dry wt. (g)
TI04	26.25 ± 1.48 a	16.65 ± 1.18 a	0.211 ± 0.034 a
TI15	24.90 ± 1.90 ab	15.30 ± 1.21 ab	0.186 ± 0.024 ab
TI19	23.92 ± 1.91 abc	14.20 ± 1.09 bc	0.169 ± 0.019 bc
TI29	24.20 ± 1.03 ab	14.40 ± 1.26 bc	0.172 ± 0.012 bc
T10	25.10 ± 1.49 ab	14.75 ± 1.14 abc	0.180 ± 0.019 bc
Tri1	24.10 ± 1.98 abc	14.10 ± 1.70 bc	0.168 ± 0.015 bc
THP2	23.05 ± 1.55 bc	13.35 ± 1.60 bc	0.163 ± 0.019 bc
JF17-3	24.30 ± 1.81 ab	13.95 ± 1.19 bc	0.172 ± 0.023 bc
TD18	23.80 ± 1.48 bc	13.70 ± 1.36 bc	0.165 ± 0.023 bc
SI	24.25 ± 1.02 ab	14.42 ± 1.23 bc	0.171 ± 0.010 bc
Control	21.75 ± 1.03 c	12.85 ± 1.94 c	0.154 ± 0.013 c

Data (means ± SE, *n* = 5) in the same column with different letters are significantly different at *p* < 0.05 by Tukey’s HSD test or Conover test.

### Biosustained spores and SI effects in rice field’s conditions with accumulated *Eucalyptus* leaves

**Figure 7:**
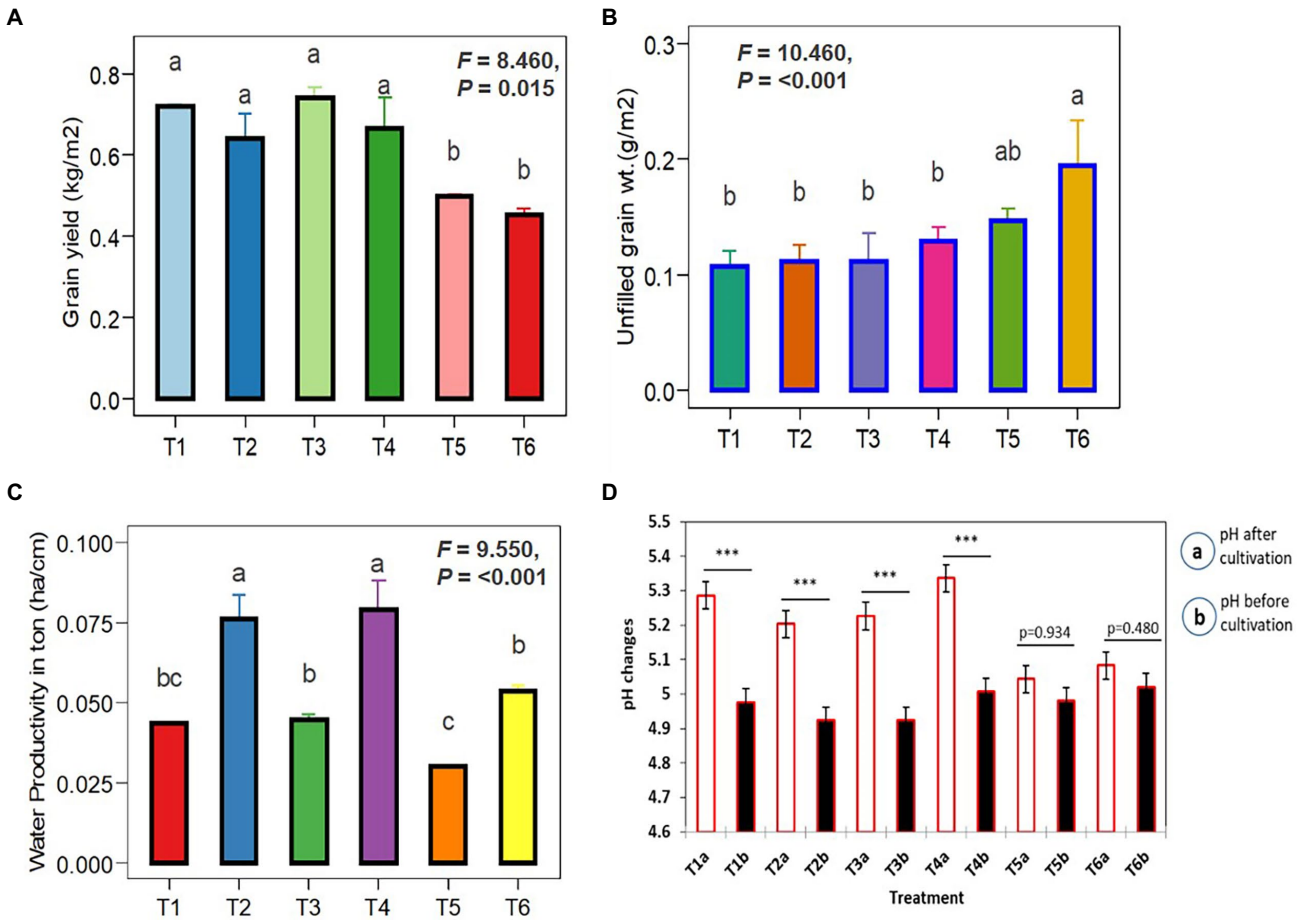
One-way ANOVA illustrates the influence of *Trichoderma* spores with SI under different cultivation conditions. **(A)** Filled grain yield per m^2^. **(B)** Unfilled grain yield per m^2^. **(C)** Water productivity under CP (T1, T3, and T5) and AFW (T2, T4 and T6). **(D)** pH changes before and after inoculation (*Trichoderma* + SI) was compared by pairwise comparison (ns *p* ≥ 0.05, *p < 0.05, **p < 0.01, ***p < 0.001). Data are means ± SD (*n* = 5). Bars without shared letters indicate significant differences according to Tukey’s HSD test.

Figure 7 depicts the effects of *Trichoderma* (TI04 and TI15) and SI on rice yield and soil pH. Grain yields in treated plots were significantly higher than in control plots. However, compared to the control, the yield per square meter increased significantly in T1-TI04 (CP: continuous ponding), T2-TI04 (AFW: alternate flooding and wetting at 1 day after the disappearance of 4 cm), T3-TI15 (CP), and T4-TI15 (AFW). In the case of irrigation difference (CP-AFW), grain yield increased by 9.35% in CP (control), 11.08% in TI04, and 10.43% in TI15, respectively. All treatments have a threefold increase in yield from control, with T1 yields rising by 30.79%, T2 by 29.45%, T3 by 32.72%, and T4 by 31.91%. The unfilled grain yield was decreased by an average of 26.75% in T1, 42.73% in T2, 23.58% in T3, and 33.84% in T4 treatments as compared to control. T1, T2, T3, and T4 all demonstrated a slight pH increase as compared to the control, with T2 and T4 showing a greater increase under AFW conditions. In the term of water productivity, it was determined that the irrigation water applied for treatments CP and AFW was 164 cm and 84 cm, respectively. Water productivity (t ha^−1^ cm^−1^) among the treatments receiving irrigation water was found to be lowest (0.0301) in treatment T5, and highest in treatments T2 and T4 with 0.0763 and 0.0791, respectively. According to these findings, there was a significant difference between CP and AFW, and water productivity declined as irrigation water use increased (Figure 7).

**Figure 8:**
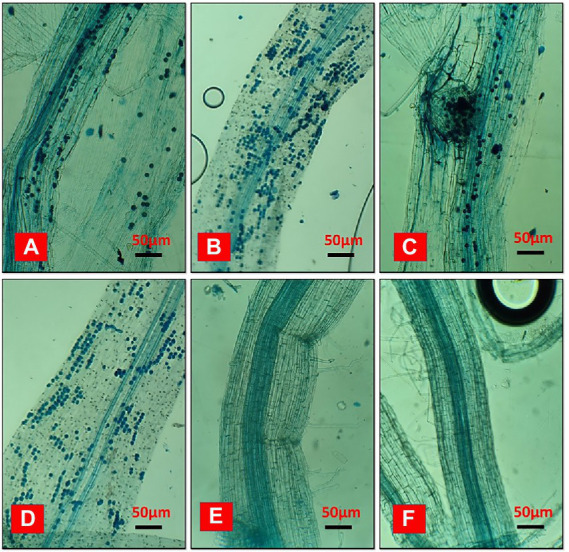
The effects of biosustained spores of *Trichoderma* isolates on the colonization of *Serendipita indica* in rice roots under accumulated field conditions of *Eucalyptus* leaves. **(A)** T1-TI04 (78.12%); **(B)** T2-TI04 (86.36%); **(C)** T3-TI15 (77.22%); **(D)** T4-TI15 (83.16%); **(E)** T5-Control (0%); **(F)** T6-Control (0%).

In a field ecosystem, rice roots were effectively infected with *Serendipita indica*, but relative colonization rates varied significantly between treatments (Figure 8). According to the findings, irrigation and the degradation capability of *Trichoderma* isolate had a significant impact on the SI colonization rate of rice roots. The rates of root colonization varied significantly between treatments, with T2 and T4 having the highest rates of colonization at 86.36% and 83.16%, respectively, whereas T1 and T3 root colonization rates were 78.12% and 77.22%, respectively. Furthermore, the T2 and T4 treatments produced more chlamydospores than the T1 and T3 treatments. On the other hand, no colonization was found in control. Unfilled grain yield was affected by AFW conditions, but this was reduced in treated plants due to the effect of *Trichoderma* and SI applications because it is evident that they can alleviate draught effects on plant growth by providing draught-resistant capacity to plants. Extracellular alkalinization has been observed in a variety of fungal species under certain conditions. One of them is carbon deficiency, which occurs in low-glucose conditions (Vylkova et al., 2011; Abuhena et al., 2022b). The nutrient starvation method produced bio-sustained spores that improved rice yield and soil pH, indicating that the spores’ agronomic character remained unchanged.

## Discussion

*Eucalyptus* leaf degradation by microbial inoculum provides a two-fold advantage: one for ecosystem restoration and the other for agriculture yield improvement by increasing nutrient availability. Because of the various ecological hazards created by *Eucalyptus*, which include drops in groundwater level and biocidal activity of accumulated leaves, they pose an unknown danger to soil ecosystems, where microbial processing is necessary for rapid degradation of the leaves (Abuhena et al., 2022a). This process is crucial from the perspective of world food security and environmental sustainability because of the large volume of waste materials that are generated and can cause environmental hazards. All the waste produced by agro-industrial developments as well as unethical human activities can be processed into beneficial products by using microorganisms (Sadh et al., 2018). Microbes available on-site can meet the microbial inoculum requirements of the agricultural areas. It will minimize the impending hazards in the food sector and ecosystem restoration. The active agent of microbial inoculum should have waste-degrading capacity along with several plant growth-promoting features for application under these conditions (Abuhena et al., 2022c). In this study, we isolated *Trichoderma* strains from different agroecological zones from samples like decaying wood and rhizosphere soil, based on indications that the isolates may be good degraders and beneficial for plants. Bioactivity assays revealed that the isolates produce IAA, solubilize phosphate, secrete chitinase and β-1,3-glucanase, and show antagonism against phytopathogens such as *Sclerotium theobromae*, *Curvularia spicifera*, *Alternaria alternate*, and *Fusarium equiseti* (Abuhena et al., 2022c).

Since microorganisms have a wide range of physiological properties, nutrients have a significant impact on their growth. In addition to nutrients, other factors such as pH, temperature, and aeration are also important for *Trichoderma* growth. The growth structures of *Trichoderma*, such as hyphae, mycelia, chlamydospore, and spore, as well as the techniques of production, all have an impact on their potential (Hewavitharana et al., 2018). The LAISS method, which is discussed in this article, was developed to be used for the production of *Trichoderma* spores that can endure stressful situations including low pH and nutrient scarcity. pH and nutrition seem to be the most crucial variables, according to the results of using this technique. Lower pH and nutritional stress (WA) led to the production of more spores with higher densities, whereas normal growth circumstances in PDA resulted in the production of fewer spores with lower densities. The quantity, density, pureness, and sustainability of spores are important considerations when choosing a production strategy. In this research, LAISS was found to be a suitable strategy for spore production, most possibly owing to the presence of limited soluble sugar, nitrogen content, fiber, and starches in WA that *Trichoderma* utilizes (Mohamed et al., 2011). The quantity, density, and purity of the concentrated spore (1.38 × 10^11^ CFU/g) produced by the LAISS were noticeably better than those produced by conventional substrate-based SSF (Sachdev et al., 2018; Naeimi et al., 2020; Mulatu et al., 2021). Spores with a high initial moisture content must be dried to a low moisture level. On the other hand, rapid drying will result in a certain proportion of cells dying (Jin and Custis, 2011). The method employed in this study resulted in distinctive biosustained spores without the need for drying to change the moisture content. This method conserved the spore’s vitality by preventing contamination, which also decreases downstream spore viability. Previous studies have demonstrated that dry and aerial spores are significantly more bio-sustainable, which supports and confirms the utilization of spore culture on WA in this method (Naeimi et al., 2020; Mulatu et al., 2021).

*Eucalyptus* trees have very hazardous effects on the ecosystem and crop yields due to their growth pattern and toxic leaves. *Eucalyptus* leaves are decomposed by beneficial fungi, that turn the hazardous leaves into organic matter. The benefits of greater organic matter in the soil include a better environment for soil organisms, a reserve of plant nutrients, improved soil structure that encourages root growth, and improved aeration. Numerous research claims that *Eucalyptus* trees produce large amounts of litter that is rich in lignin, phenol, and C:N ratios (Castro-Díez et al., 2012). The benefits of *Trichoderma* for composting have been extensively researched. According to reports, *Trichoderma harzianum* inoculation of straw-based compost piles resulted in a composting time that was less than half that of traditional approaches (Cuevas, 1997). Another study found that treating rice straw with *Trichoderma harzianum* and cow dung slurry increased the population of beneficial bacteria and increased crop yield (Sannathimmappa et al., 2015). The decomposition times of empty fruit bunches and palm oil mill effluent were said to have been reduced from 4 to 6 months to 21 to 45 days (Amira et al., 2011). In this study, we investigated the biosustainability, growth pattern, and degrading potential of various *Trichoderma* isolates on *Eucalyptus* leaves. Although all of the isolates were grown on *Eucalyptus* leaves, their growth patterns and degrading potential varied. With spore densities of 5.26 × 10^8^ (CFU/g) and 7.05 × 10^7^ (CFU/g), respectively, strains TI04 (*Trichoderma viride*) and TI15 (*Trichoderma citrinoviride*) demonstrated the highest growth on leaves. Isolates TI15 and TI04 both showed leaf weight losses of 31.33% and 26.8% after 15 days of incubation, respectively. Pozo, Cortes et al., and Canhoto reported that E. globulus lost 50% of its weight in 30–50 days, 76 days, and 84 days, respectively (Pozo, 1993; Cortes et al., 1994; Canhoto and Graca, 1995). The majority of the decomposing process did not investigate the bio-sustainability and spore density of *Trichoderma* on degraded leaves. But according to Organo et al., rice straw had the highest cell density (1.3 × 10^5^ CFU/g), which also confirmed the presence of contaminants (Organo et al., 2022). In this present experiment, excellent sporulation (5.26 × 10^8^ CFU/g), with no contaminants detected on the leaf surface or in the harvested degraded materials, was achieved.

In a rice seedling experiment, we used *Serendipita indica* and biosustained *Trichoderma* spores from degraded *Eucalyptus* leaves. The plant-friendly properties of the strain, such as phosphate solubilization, IAA production, cell wall degrading enzyme production, and siderophore synthesis, benefited rice seedlings. The growth of rice seedlings in terms of shoot length (cm), root length (cm), and biomass (g) was improved by strains TI04 and TI15. Moreover, the microbial community, nutrients, and soil texture all have an impact on *Serendipita indica* colonization (Dias et al., 2020). In our study, *Trichoderma* spores with degraded leaves enhanced SI mycorrhizae’s root association. In seedlings treated with TI15 and TI04, the rates (percentage) of SI colonization were 88.42 and 84.75, respectively. This represents an increase of 12.9% and 8.3%, respectively, over the sole use of SI. *Trichoderma* and mycorrhizae are widely utilized in agriculture as advantageous microbes, but little is documented about the molecular variations in the roots when these fungi are combined. According to reports, applying *Trichoderma* and mycorrhizae together improves the nutritional content of a variety of plants, including cucumbers, tomatoes, and melon (Poveda et al., 2019). However, when these two fungi are combined, adverse impacts on plants have also been reported (Poveda et al., 2019). However, this will be the first time that it has been documented that *Serendipita indica* colonization was increased by the application of *Trichoderma* spores with degraded leaves, which may aid in the restoration of the soil microbial community in degraded ecosystem.

In the current study, *Trichoderma* biosustained spores (TI04 and TI15) significantly increased rice yield under both CP and AFW conditions. Additionally, the SI colonization improved in field conditions (accumulated *Eucalyptus* leaf) despite facing low pH stress and draught stress. According to different articles, Cao et al. observed lower soil pH levels in *Eucalyptus* spp. plantations, ranging from 4.2 to 4.5, whereas Alemie detected decreased soil pH ranging between 3.5 and 4.0 under *Eucalyptus* spp. trees (Cao et al., 2010; Alemie et al., 2013). As soil biological activities are decreased in acidic soils, Castro-Dez et al. found that low soil pH restricts the growth and activity of decomposing soil microorganisms (Mohamed et al., 2011). According to Berendse, soil trace elements including manganese (Mn) and aluminum (Al) become hazardous for the majority of plant growth at pH values below 5.5 (Berendse, 1998). Additionally, in acidic soils, nutrients like phosphorus and nitrogen usually combine with Al and Fe to produce insoluble compounds that become adsorbed and are inaccessible to plants.

In this study, the risks associated with an acidic pH were reduced because it has been noted that a variety of fungal species can alkalinize their extracellular matrix under specific circumstances. One of these is carbon deficiency, which occurs when there is little glucose (Vylkova et al., 2011). According to Rosado et al., the cell wall protein qid74, chitinase, protease, and glucose permease are among the *Trichoderma* genes that are affected by pH in various circumstances (Rosado et al., 2007). One of these is carbon deficiency, which occurs when there is little glucose (Vylkova et al., 2011). According to Rosado et al. (2007), the cell wall protein qid74, chitinase, protease, and glucose permease are among the *Trichoderma* genes that are affected by pH in various ways. PacC specifically lowers glucose permease and protease expression in *T. harzianum* while increasing chitinase and qid74 expression. Inhibitory metabolite accumulation is also prevented by PacC. *Trichoderma* does so by extracellular alkalization during sporulation, which raises the pH of the substrate. FA was likewise employed to modify soil pH.

On the other hand, soil samples from *Eucalyptus* spp. plantation soils contained low quantities of organic matter (1%), according to Shamsher et al. (2002). In comparison to the area where *Eucalyptus* spp. were absent, soil total N decreased as *Eucalyptus* grew, according to Tererai et al. (2014). Tererai et al. also provide an explanation for why the resistant litter and consequently poor breakdown rates of the leaves result in lower average soil total N, P, and K concentrations in *Eucalyptus* spp. plantation soils (Tererai et al., 2014). From this perspective, extracellular enzymes from *Trichoderma*, such as cellulases, are critical in decomposing organic polymers into simpler forms that can be transported inside the cell, and since *Trichoderma* is known to produce large amounts of cellulolytic and hemicellulolytic enzymes, it is attributable that inoculated treatments contain more cellulases than uninoculated treatments. This will increase the break-down of the cellulosic substrates in the *Eucalyptus* leaves, resulting in the production of more available nutrients that will promote fungal growth.

According to Lane et al. the expansion of *Eucalyptus* spp. plantations significantly reduces groundwater levels and decreases the amount of water available for irrigation on lands that were formerly used for arable crops or occupied by grass because of the plant’s profound and heavy root system and the properties of the soil (Lane et al., 2004) This tree creates draught concerns in the northern areas of Bangladesh since *Eucalyptus* plantations are currently so prevalent that they may be seen in great numbers on agricultural land, nearly every yard, office building, by the side of the road, and along railroad tracks (Supplementary Figure 3). Afforestation of exotic *Eucalyptus* spp. plantations is said to have a negative impact on the soil’s physical and chemical qualities as well as the biodiversity of the plant community, according to Wen et al. (2009). In our study, in condition AFW, rice yield was not affected by the dry condition due to the effect of *Trichoderma* application since it is evident that *Trichoderma* can alleviate the draught effects on plant growth by providing draught-resistant capacity to plants. Furthermore, *S. indica* colonization improves crop growth, yield, and drought tolerance. Under drought conditions, *S. indica* regulates phytohormone signaling, photosynthesis, wax biosynthesis, the cytoskeleton, and autophagy. Stress-induced ROS are detoxified by stimulating the host plant’s antioxidant system (Jangir et al., 2021). In our study, under AFW, TI04, and TI15 had the highest rates (percentages) of SI colonization, with 86.36 and 83.16, respectively. Likewise, water productivity (t ha^−1^ cm^−1^) values for TI04 and TI15 were greater under AFW, at 0.0763 and 0.0791, respectively. The increased mycorrhizae networking is an indicator of drought stress alleviation of rice plants and promotes the existing microbial community restoration.

This study’s findings revealed that manufacturing is an important stage in the development of a fungal inoculum, with high biosustainability and synergistic application of the inoculum, all of which contribute to the success of practical novel application. The inoculum’s efficacy in novel applications such as hazard mitigation and yield improvement in stressed conditions is a good indicator of its environmental usefulness. In this regard, the current study provides evidence for widespread use of this approach to reduce hazards and restore the agricultural ecosystem.

## Conclusion

Overall, the findings demonstrate that *Trichoderma* could be grown using the nutrient deprivation technique at low pH to obtain biosustained spores. The biosustained spores had the enormous capacity to breakdown *Eucalyptus* leaves *in vitro* in a substantially shorter time than under normal circumstances. The biosustained spores and mycorrhizae were found to be a surprisingly effective combination in the field for decomposing *Eucalyptus* leaves, reducing water scarcity and low-pH risks created by *Eucalyptus* trees in crop production and restoring the existing soil ecology.

## Data availability statement

The original contributions presented in the study are included in the article/Supplementary material, further inquiries can be directed to the corresponding authors.

## Author contributions

MGK, MA, and MFA: conceptualization of the research work, performer of the experimental research work, data collection, statistical analysis, manuscript writing, manuscript revision, and editing. JA-R: data collection, performer of the experimental research work, manuscript writing, reviewing, and editing. NMR: manuscript writing, reviewing, editing and, approval of the protocol. DM and PM: statistical analysis, manuscript writing, reviewing, and editing. YW: manuscript writing, reviewing and submission of the manuscript. All authors contributed to the article and approved the submitted version.

## Funding

This study was supported by Apex Biofertilizers & Biopesticides Limited, Gobindaganj-5740, Gaibandha, Bangladesh, Apex Biotechnology Laboratory, Apex Holdings Ltd., East Chandora, Shafipur, Kaliakoir, Gazipur 1751, Bangladesh and the Initial Scientific Research Fund of Baotou Teachers’ College, Inner Mongolia, China (Grant no. BTTCRCQD2020-001).

## Conflict of interest

MGK, MA, MFA, JA-R, and NMR were employed by Apex Biofertilizers and Biopesticides Limited. MA, NMR, and PM were employed by Apex Holdings Ltd.

The remaining authors declare that the research was conducted in the absence of any commercial or financial relationships that could be construed as a potential conflict of interest.

The authors declare that this study received funding from Apex Biofertilizers & Biopesticides Limited and Apex Holdings Ltd. The funder had the following involvement in the study: The funder provided overall guidance for the design of the study, conduct of the experiment, data collection, analysis, and interpretation, as well as the decision to submit this work for publication.

## Publisher’s note

All claims expressed in this article are solely those of the authors and do not necessarily represent those of their affiliated organizations, or those of the publisher, the editors and the reviewers. Any product that may be evaluated in this article, or claim that may be made by its manufacturer, is not guaranteed or endorsed by the publisher.

## Footnotes

^1^ http://www.ncbi.nlm.nih.gov
