# Supplementary material for: A bio-sustainable approach for reducing Eucalyptus tree-caused agricultural ecosystem hazards employing Trichoderma bio-sustained spores and mycorrhizal networks
Source: Front Microbiol. 2023 Jan 16;13:1071392. doi: 10.3389/fmicb.2022.1071392 (PMC9885803; doi:10.3389/fmicb.2022.1071392)
Supplement: Supplementary file 1 [file Data_Sheet_1.docx]

**Supplementary Information for**

A bio-sustainable approach for reducing *Eucalyptus* tree-caused agricultural ecosystem hazards employing *Trichoderma* bio-sustained spores and mycorrhizal networks

Md. Golam Kabir^1,2,3†^, Yonglong Wang*^4^, Md. Abuhena*^1,5†^, Md. Faisal Azim^1†^, Jubair Al-Rashid^1,3,6^, Noorain Munim Rasul^1,5^, Dipa Mandal^7^ and Pulak Maitra*^5,8^

^1^Department of Research & Development, Apex Biofertilizers & Biopesticides Limited, Gobindaganj-5740, Gaibandha, Bangladesh

^2^Institute of Soil Science, Chinese Academy of Sciences, Nanjing, 210008, China

^3^University of Chinese Academy of Sciences, Beijing, 100049, China

^4^Faculty of Biological Science and technology, Baotou Teacher's College, Baotou, Inner Mongolia, China

^5^Apex Biotechnology Laboratory, Apex Holdings Ltd., East Chandora, Shafipur, Kaliakoir, Gazipur 1751, Bangladesh

^6^Tianjin Institute of Industrial Biotechnology, Chinese Academy of Sciences, Tianjin, 300308, China

^7^Institute of Microbiology, University of Chinese Academy of Sciences, Beijing 100101, China

^8^Institute of Dendrology, Polish Academy of Sciences, Kornik-62035, Poland

† These authors contributed equally to this work

**Supplementary Tables 1-2**

**Supplementary Figures 1-4**

**Supplementary Tables**

**Supplementary Table 1.** Morphological characterization of *Trichoderma* isolates using plates (PDA) culture and microscopy.

| **Isolate** | **Species identified** | **Colony** | | | **Mycelia** | | **Conidia** | | | | **Phialides** | | |
| --- | --- | --- | --- | --- | --- | --- | --- | --- | --- | --- | --- | --- | --- |
|  |  | **Colour** | **Reverse**  **colour** | **Edge** | **Form** | **Colour** | **Conidiation** | **Branching** | **Shape** | **Size (μm)** | **Shape** | **Size (μm)** | **Disposition** |
| TI04 | *Trichoderma viride* | Whitish deep green | Light green | Zigzag | Floccose | White | Concentric Zones | Irregularly branched; branches usually not paired | Globose | 2.9-3.2 | Slender | 2.5-11.2 | Clustered |
| TI15 | *T. citrinoviride* | Yellowish Green | Yellow | Smooth | Floccose | White | Concentric Zones | Highly Branched | Ellipsodal, Obovoid | 2.5-3.4 | Nine-pin shape | 3.2-12 | Tending clustered, 2-3 whorls |
| TI19 | *T. reesei* | Yellowish Green | Yellowish | Zigzag | Floccose | Watery White | Concentric Zones | Highly Branched, Irregular | Ellipsodal, obovoid, | 2.6-3.7 | Nine-pin shape | 2.7-12.5 | Tending clustered, 2-3 whorls |
| TI29 | *T. asperellum* | Deep Green with whitish patch | Light Green with whitish patch | Smooth | Floccose | White | Inconspicuous | Regularly branched; branches typically paired | Globose to subglobose | 1.3-3.7 | Slender | 3.5-11.3 | Clustered |
| T10 | *T. longibrachiatum* | Yellowish deep green | Yellow | Slightly wavy | Floccose | White | Ring like zone | Irregularly Branched | Ellipsodal, obovoid, dilute green | 2.2-3.3 | Bowling pin, lageniform | 4.2-7.6 | Solitary |
| Tri1 | *T. hamatum* | Whitish deep green | Whitish | Zigzag | Floccose | White | Ring like zone | Highly branched | Ellipsoidal | 1.5-3.4 | Nine-pin shape |  | Densely clustered |
| THP2 | *T. asperellum* | Deep Green with Whitish patch mix | Whitish | Zigzag | Floccose | Watery White | Concentric Zones | Regularly Branched, branches are typically paired | Subglobose or ovoidal | 1.4-3.9 | Straight | 3.2-11.9 | Cruciate whorls |
| JF17-3 | *T. asperellum* | Deep Green | Greenish | Smooth | Floccose | White | Concentric Zones | Moderately branched | Subglobose or ovoidal | 1.3-3.7 | Nine-pin shape | 2.7-12.6 |  |
| TD18 | *T. longibrachiatum* | Yellowish Green | Yellowish | Smooth | Floccose | Watery White | Concentric rings | Highly Branched | Ellipsodal, obovoid, dilute green | 2.7-3.8 | Nine-pin shape | 4.2-7.5 | Solitary |

**Supplementary Table T2.** List of *Trichoderma* isolates and their host or sources, location, identification and gene bank accession number.

| Sl. No. | Isolate Code | Sample name | Sample Location | Coordination | Species identified | Accession number |
| --- | --- | --- | --- | --- | --- | --- |
| 1 | TI04 | Decayed wood of rain tree | Banaripara, Barishal | 22°48'47.5"N 90°08'03.7"E | *Trichoderma viride* | KX495149 |
| 2 | TI15 | Akashmoni tree dry wood | Kaliakoir, Gazipur | 24°03'27.3"N 90°15'53.8"E | *T. citrinoviride* | MH084943 |
| 3 | TI19 | Eucalyptus tree dry wood | Kaliakoir, Gazipur | 24°02'40.7"N 90°15'57.0"E | *T. reesei* | MH084948 |
| 4 | TI29 | Rice Rhizosphere Soil | Kaliakoir, Gazipur | 24°03'55.2"N 90°16'03.9"E | *T. asperellum* | MK478868 |
| 5 | T10 | Tomato Rhizosphare | Kaliakoir, Gazipur | 24°02'10.3"N 90°15'58.5"E | *T. longibrachiatum* | KX495150 |
| 6 | Tri-1 | Grass root | Kaliakoir, Gazipur | 24°02'13.5"N 90°15'57.3"E | *T. hamatum* | KX495148 |
| 7 | THP2 | Gub tree Rhizosphere soil | Pirojpur Sadar | 22°36'37.7"N 89°59'34.5"E | *T. asperellum* | MH091328 |
| 8 | JF17-3 | Jute Rhizosphere soil | Saltha, Faridpur | 23°29'14.7"N 89°47'51.0"E | *T. asperellum* | MH091329 |
| 9 | TD18 | Rotten mushroom compost | Savar, Dhaka | 23°51'01.4"N 90°15'28.2"E | *T. longibrachiatum* | MH084968 |

**Supplementary Figures 1-4**


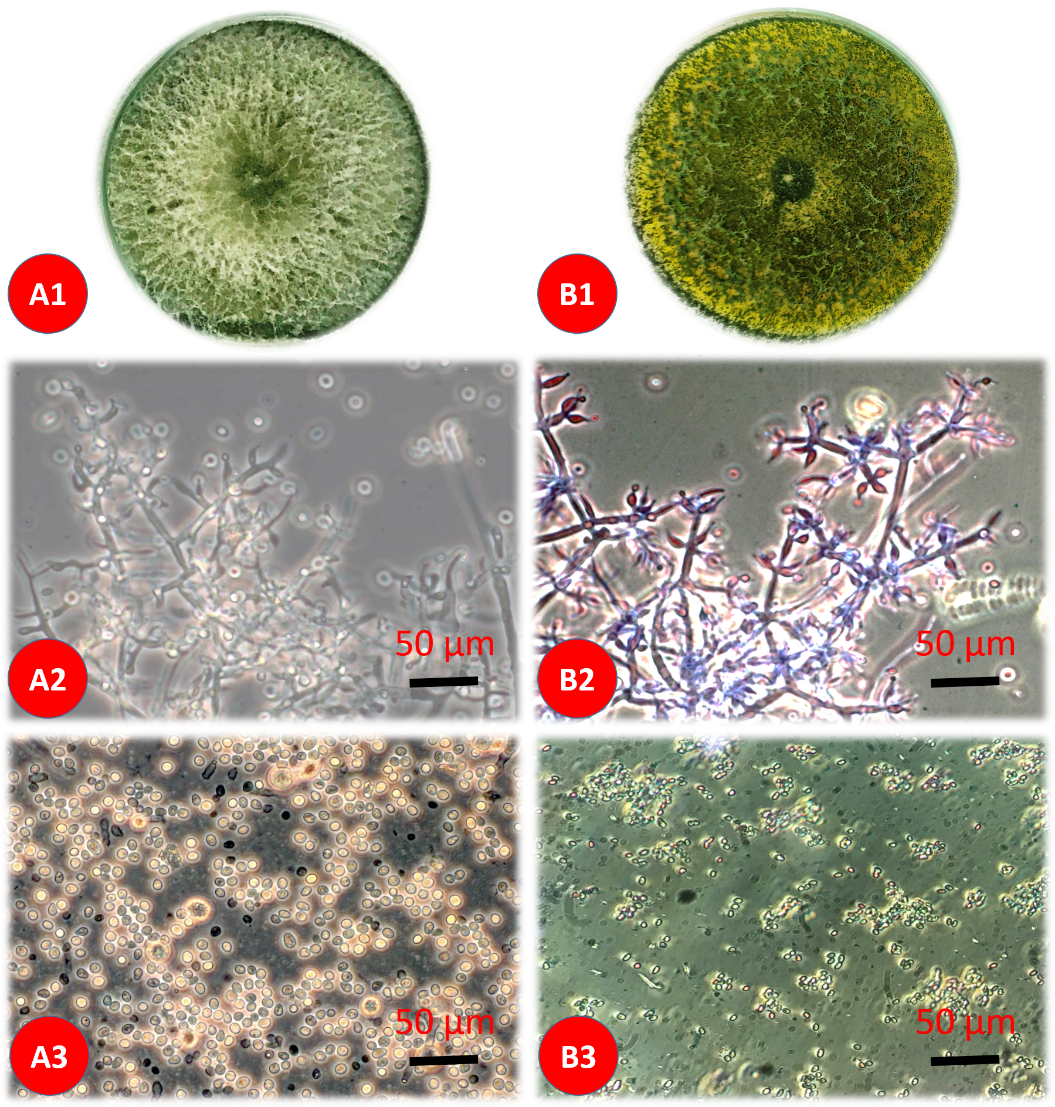


Supplementary Figure 1. Morphology of *Trichoderma* spp. (A1, A2 & A3) The growth of *Trichoderma viride* TI04 on a PDA plate after 5 days of incubation and the morphology of mycelia and spores under a phase-contrast microscope (Axio Imager A1, Carl Zeiss, Germany) at 40X. (B1, B2 & B3) The growth of *T. citrinoviride* TI15 on a PDA plate after 5 days of incubation and the morphology of mycelia and spores under a phase-contrast microscope.

**
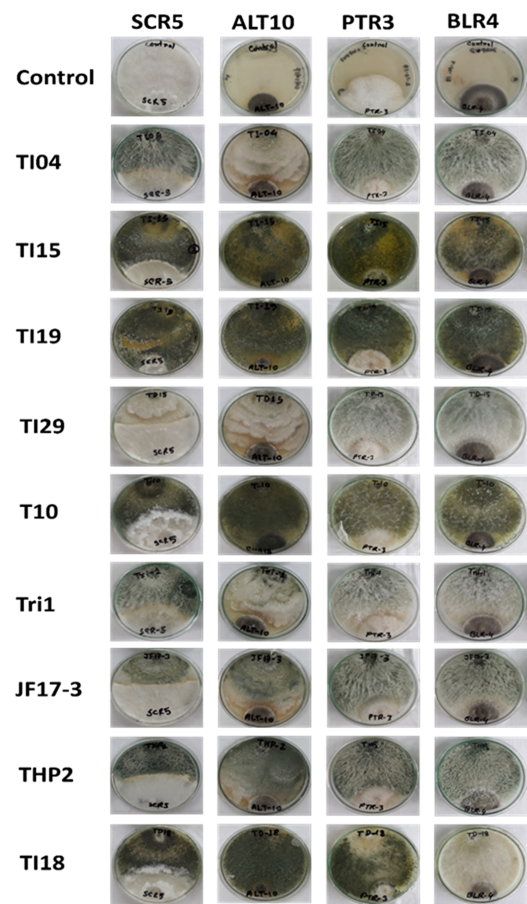
**

**Supplementary Figure 2.** Antagonistic activity of nine *Trichoderma* isolates against four phytopathogens: *Sclerotium delphinii* SC5*, Fusarium equiseti* PTR3*, Curvularia spicifera* BLR4, *and Alternaria alternata* ALT10*.*

*
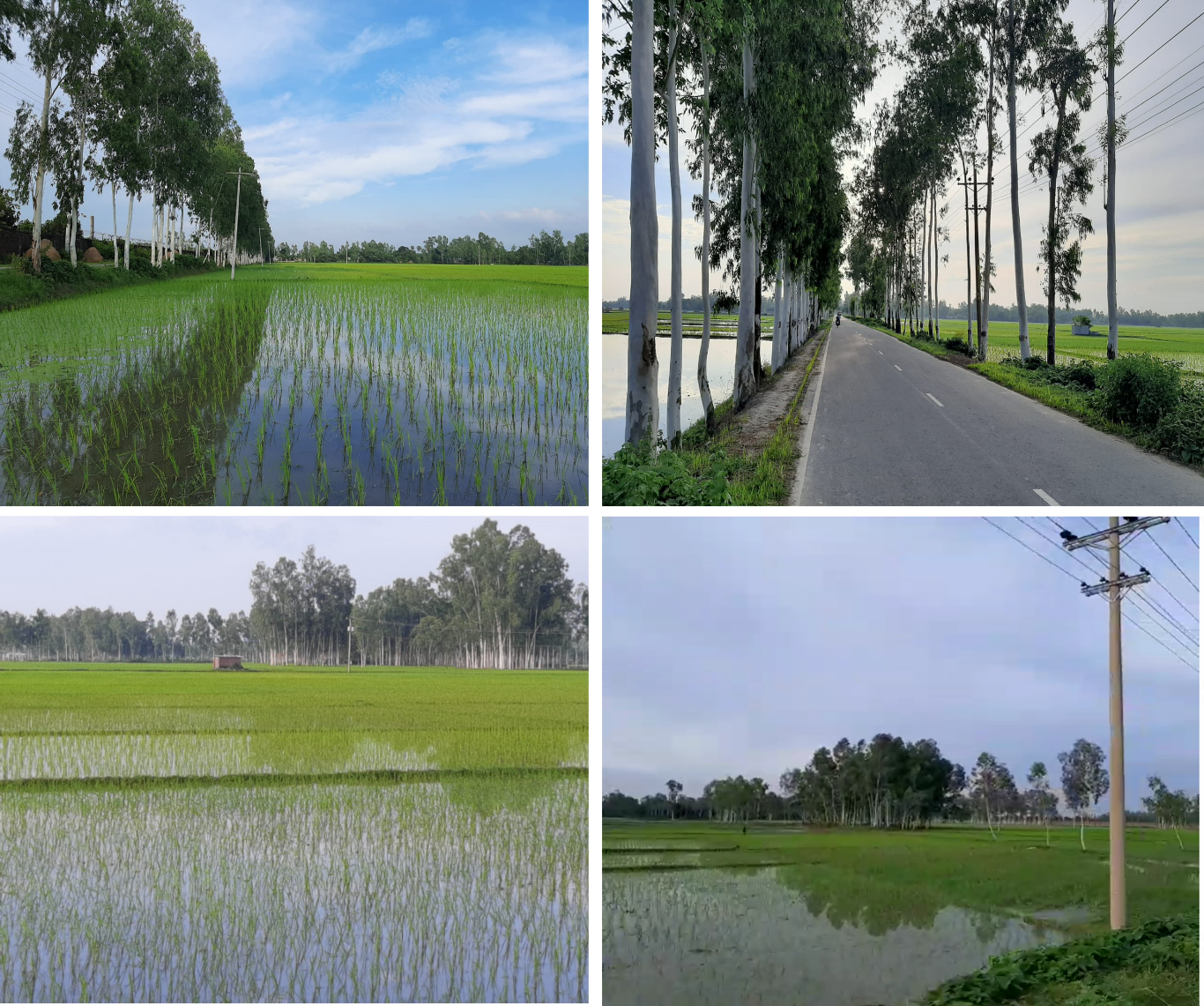
*

**Supplementary Figure 3.** Distribution of eucalyptus trees in the northern region of Bangladesh.

*
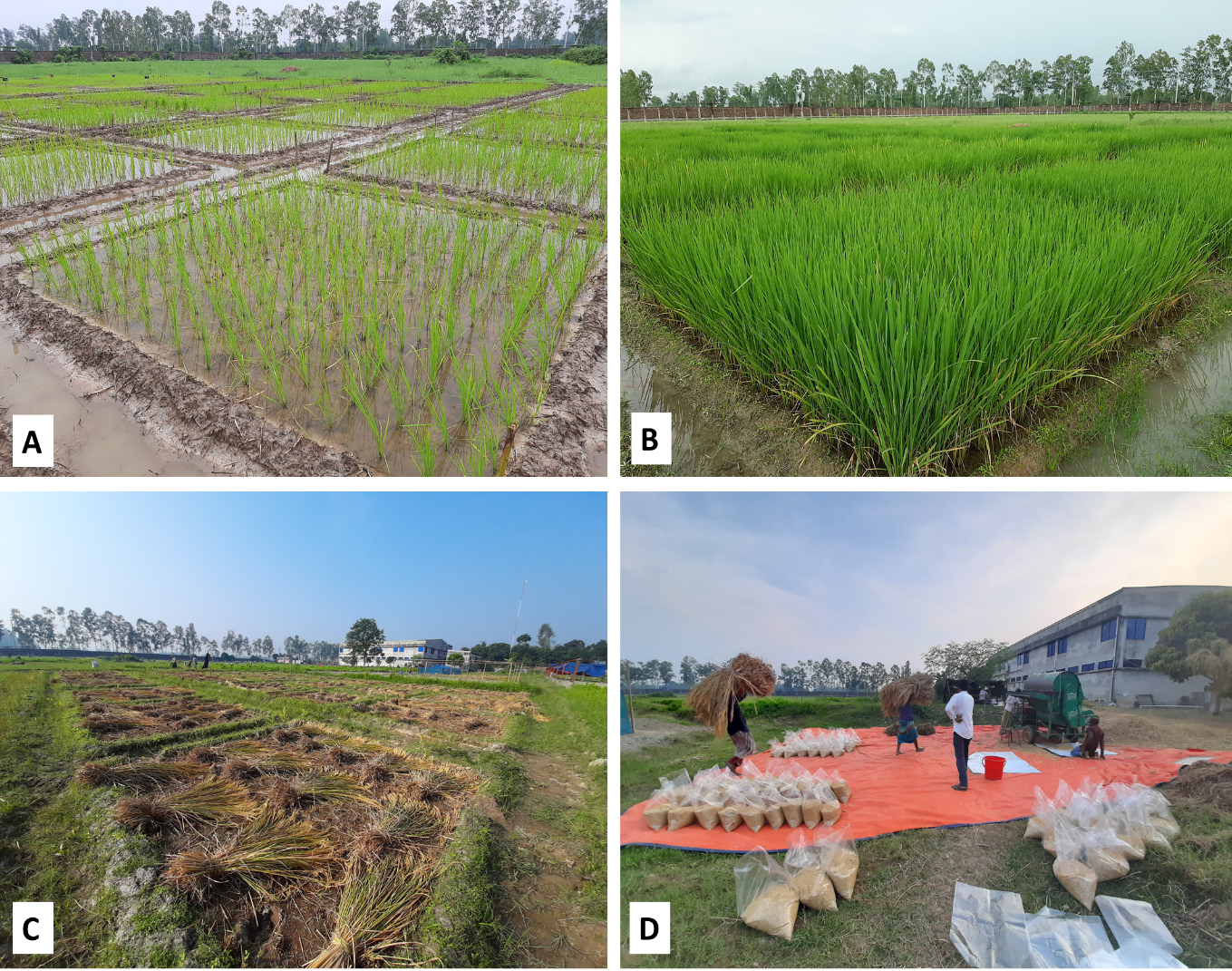
*

**Supplementary Figure 4.** Rice field experiment. A) Growth at 5 days following seedling transplantation. B) Growth at 40 days following seedling transplantation. C & D) Harvesting and data collection.
